# Radiocesium Distribution in Bamboo Shoots after the Fukushima Nuclear Accident

**DOI:** 10.1371/journal.pone.0097659

**Published:** 2014-05-15

**Authors:** Takumi Higaki, Shogo Higaki, Masahiro Hirota, Seiichiro Hasezawa

**Affiliations:** 1 Department of Integrated Biosciences, Graduate School of Frontier Sciences, The University of Tokyo, Kashiwanoha, Kashiwa, Chiba, Japan; 2 Radioisotope Center, The University of Tokyo, Yayoi, Bunkyo-ku, Tokyo, Japan; 3 Research Center for Human and Environmental Sciences, Shinshu University, Matsumoto, Nagano, Japan; 4 Advanced Measurement and Analysis, Japan Science and Technology Agency (JST), Chiyoda-ku, Tokyo, Japan; University of Vigo, Spain

## Abstract

The distribution of radiocesium was examined in bamboo shoots, *Phyllostachys pubescens*, collected from 10 sites located some 41 to 1140 km from the Fukushima Daiichi nuclear power plant, Japan, in the Spring of 2012, 1 year after the Fukushima nuclear accident. Maximum activity concentrations for radiocesium ^134^Cs and ^137^Cs in the edible bamboo shoot parts, 41 km away from the Fukushima Daiichi plant, were in excess of 15.3 and 21.8 kBq/kg (dry weight basis; 1.34 and 1.92 kBq/kg, fresh weight), respectively. In the radiocesium-contaminated samples, the radiocesium activities were higher in the inner tip parts, including the upper edible parts and the apical culm sheath, than in the hardened culm sheath and underground basal parts. The radiocesium/potassium ratios also tended to be higher in the inner tip parts. The radiocesium activities increased with bamboo shoot length in another bamboo species, *Phyllostachys bambusoides*, suggesting that radiocesium accumulated in the inner tip parts during growth of the shoots.

## Introduction

On March 11, 2011, a catastrophic earthquake and subsequent tsunami along the Fukushima coast severely damaged the Tokyo Electric Power Company (TEPCO) Fukushima Daiichi nuclear power plant, resulting in substantial release of radionuclides from the reactors. The total amount of released radionuclides has been estimated to be 520 PBq (excluding noble gases); for releases of ^137^Cs, estimations are about 12 PBq, which is about 15% of that released at Chernobyl [1]. The Fukushima-derived radiocesium (^134^Cs and ^137^Cs) largely fell on land in the Tohoku and Kanto regions of Japan [2], [3] and in the Northwest Pacific Ocean [4], [5]. Radiocesium contamination in agricultural and woody plants has been reported [6], [7], [8]. Radiocesium contamination of food crops has become of prime interest, especially for residents in the affected areas.

Bamboo is a fast growing plant owing to the speed of culm growth, being up to 3–30 m long within 3–4 months, depending on species [9]. Bamboos develop a root mat of highly efficient fine roots that are usually confined to the topmost soil layer. Natural mineralization of nutrients is quicker in the topmost soil horizon than in the deeper layers, therefore, the bamboo's shallow root system can effectively absorb nutrients [9]. Bamboo shoots are a major food source that marks the beginning of spring in east Asia, including Japan. In Japan, the shoots emerge from the ground in spring and rapidly grow into long green bamboo plants. Just after the Fukushima nuclear accident from the 17th March 2011 to the 31st March 2012 the Japanese Ministry of Health, Labour and Welfare (MHLW) set a provisional regulatory value of 500 Bq/kg (fresh weight basis) for radiocesium (^134^Cs and ^137^Cs) in vegetables and crops [10], [11]. Harvested bamboo shoots from the disaster area on 27th April 2011 exceeded the regulatory limits with a maximum activity level of 3100 Bq/kg being reported [11]. On 9th May 2011, a shipment of the contaminated bamboo shoots was retained by the MHLW [12]. This batch was possibly contaminated with radiocesium-containing fallout just after the accident. However, about 2 years after the nuclear accident, radioactivity concentrations over 100 Bq/kg FW (this value has become an updated and temporary regulatory value as of 1^st^ April 2012) were detected in new bamboo shoots collected in the affected areas and reported by MHLW on 5th April 2012 [13] and 6th March 2013 [14]. Autoradiography measurements detected radioactive granular spots on the bamboo shoot skin and litter, suggesting transfer of putative microgranules with non-ionic radiocesium being transferred from the litter to the bamboo shoot skin when the bamboo shoots sprouted through the litter [15]. However, the radiocesium distributions in the bamboo shoots are not well understood. Such distributional information would be helpful, not only in understanding radiocesium transfer in plant bodies, but also in preventing human exposure from ingestion. In this pilot survey we examine the concentration distribution of radiocesium in bamboo shoots collected throughout Japan after the Fukushima nuclear accident.

## Materials and Methods

### Ethics Statement

No specific permits were required for the described field studies: a) no specific permissions were required for accessing locations and undertaking sampling activities b) the sampling locations were not privately-owned or protected; c) the field studies did not involve endangered or protected species.

### Collection of bamboo shoots

**Figure 1:**
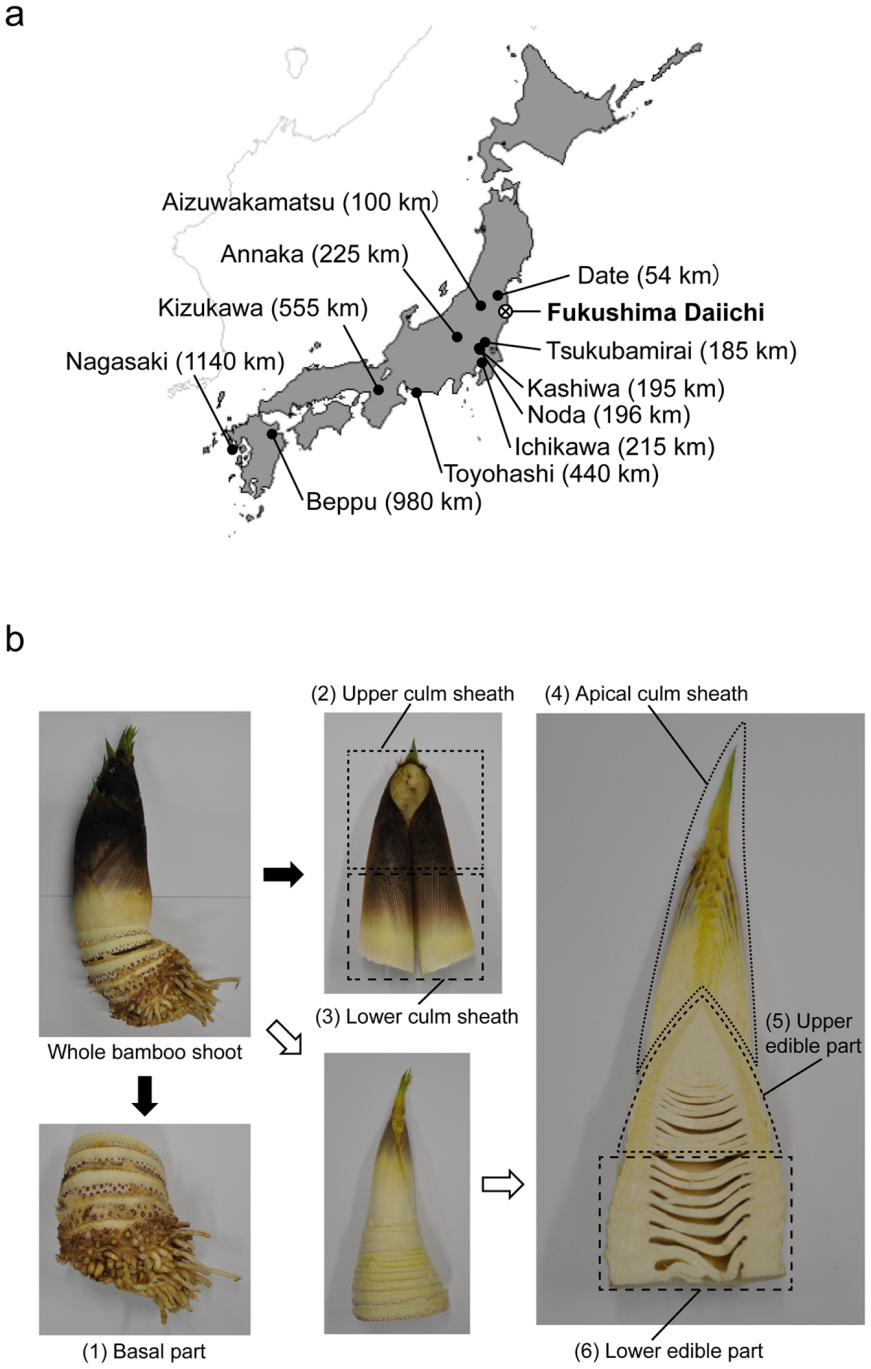
Sampling and fractionation of bamboo shoots. (a) Map of Japan showing the location of the twelve sampling sites and the Fukushima Daiichi nuclear power plant. (b) Schematic workflow of the bamboo shoot fractionation. The basal part was cut off (black down-arrow), and about 10–15 culm sheaths were stripped (black right-arrow). The culm sheaths were cut into the upper and lower parts. The stripped bamboo shoot was separated into the apical culm sheath and the upper and lower edible parts (white arrows).

Bamboo shoots of *Phyllostachys pubescens* were collected from the following locations: 1) Date, Fukushima Prefecture (41 km from Fukushima Daiichi) on 9th May 2012; 2) Aizuwakamatsu, Fukushima Prefecture (102 km from Fukushima Daiichi) on 20th May 2012; 3) Tsukubamirai, Ibaraki Prefecture (185 km from Fukushima Daiichi) on 30th April 2012 and 10th May 2013; 4) Kashiwa, Chiba Prefecture (195 km from Fukushima Daiichi) on 12th May 2012; 5) Ichikawa, Chiba Prefecture (215 km from Fukushima Daiichi) on 14th April 2012; 6) Annaka, Gunma Prefecture (225 km from Fukushima Daiichi) on 2nd May 2012; 7) Toyohashi, Aichi Prefecture (440 km from Fukushima Daiichi) on 13th April and 4th May 2012; 8) Kizukawa, Kyoto Prefecture (555 km from Fukushima Daiichi) on 14 April 2012; 9) Beppu, Oita Prefecture (980 km from Fukushima Daiichi) on 18th April 2012; 10) Nagasaki, Nagasaki Prefecture (1140 km from Fukushima Daiichi) on 9th April 2012 (Figure 1a). Bamboo shoots of *Phyllostachys bambusoides* Sieb. Et Zucc.were collected in Noda, Chiba Prefecture (196 km from Fukushima Daiichi) on 6th June 2012 (Figure 1a).

### Collection of surface soils

To evaluate the radiocesium contamination levels, surface soils (0–2 cm in depth) were collected from all the bamboo shoot sampling areas. Prior to radiocesium measurement, the soils were dried in an oven (MOV-112S; SANYO, Osaka, Japan) at 60°C for 24 hours.

### Fractionation of bamboo shoots

The bamboo shoots of *P. pubescens* were fractionated into six parts, as shown in Figure 1b. First, the (1) basal part, below the red primordial roots, with diameter of 5 mm or more, was cut off. Ten to 15 culm sheaths were stripped and cut in half, radially, to give the (2) upper and (3) lower culm sheaths. Generally, the basal part and culm sheath are not used as a food source. The stripped bamboo shoots were separated into three parts; (4) the apical culm sheath, and the (5) upper and (6) lower edible parts. After fresh weight measurement, all samples were diced and dried in an oven (MOV-112S; SANYO) at 60°C for 48 hours. The dried samples were used for dry weight and radioanalytical measurements. Moisture content was calculated on an oven-dry basis.

### Measurements of radiocesium concentrations

Each sample was placed in a polypropylene container (internal diameter 4.75 cm, height 6 cm). The samples were analyzed by gamma spectrometry, using a high purity germanium detector (IGC-30180; Princeton Gamma-Tech, Princeton, NJ, USA) and a multi-channel analyzer (DSA-1000; Canberra Industries, Meriden, CT, USA). The detector was shielded with 5 cm lead blocks and with 2 cm copper and 0.5 cm acrylic plates to reduce background contributions. The activities were determined using a standard radiation volume gamma-ray source (MX033U8PP; Japan Radioisotope Association, Tokyo, Japan). For determination of ^134^Cs activity concentrations, gamma-ray energies of 604.70 and 795.85 keV were used. The ^137^Cs activity concentrations were determined from the 661.66 keV peak energies. The counting time for each sample was 10,800 seconds and the detection limits for ^134^Cs and ^137^Cs were 0.2 Bq. The activities were determined with half-lives corrected for the dates of sampling.

### Measurement of potassium concentrations

Three grams of the diced and dried bamboo shoot samples were added to 400 mL of 1% hydrochloric acid solution, and the solutions were mixed for 30 min at room temperature. The concentrations of potassium in the solutions were measured with a flame atomic absorption spectrometer (Z-5000; Hitachi, Tokyo, Japan).

## Results

**Figure 2:**
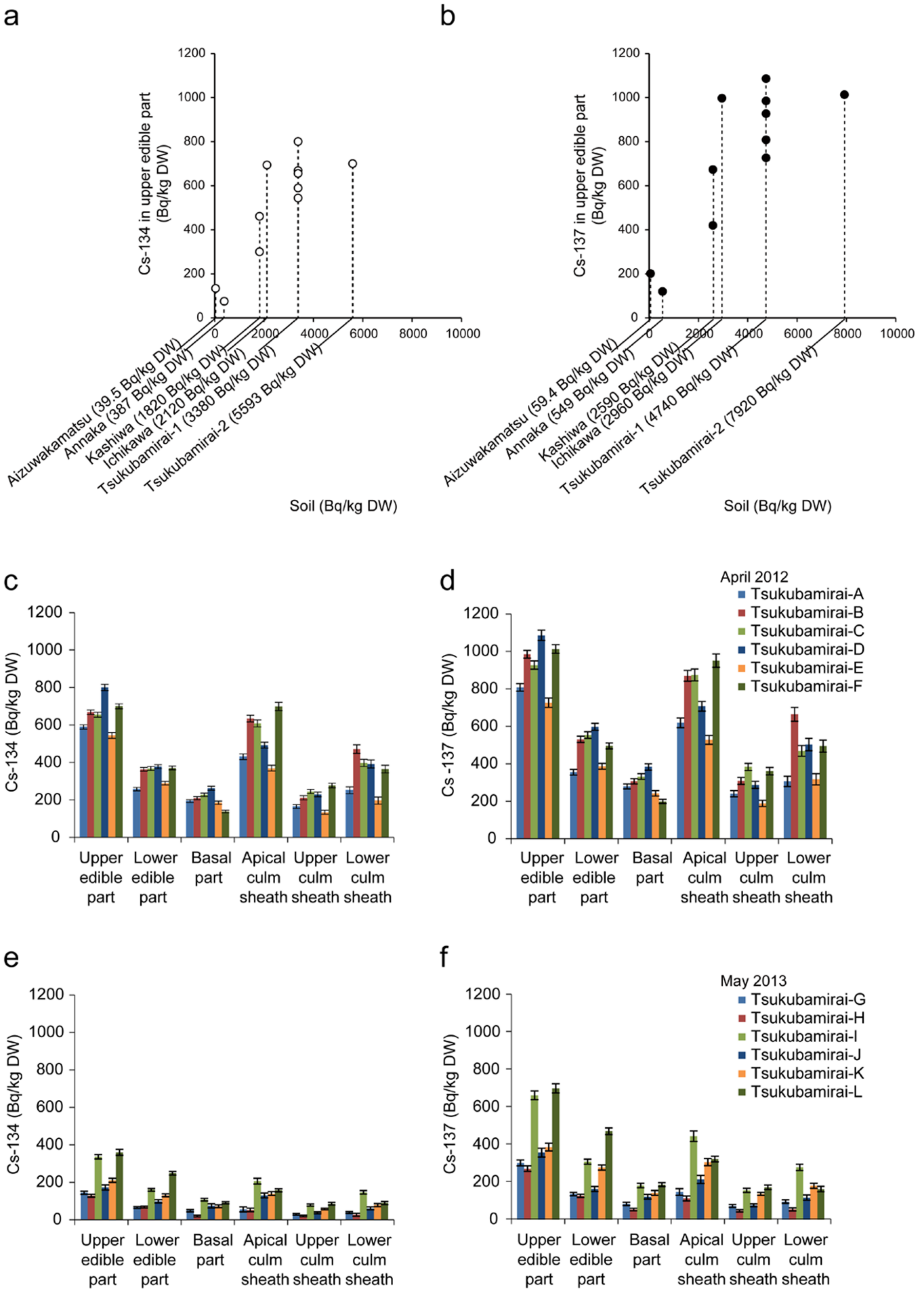
Radiocesium contamination in bamboo shoots of *Phyllostachys pubescens*. (a, b) Scatter plots of the radioactive concentrations of radiocesium, ^134^Cs (a) and ^137^Cs (b), of the surface soils and the upper edible part. (c, d) The radioactive concentrations of radiocesium, ^134^Cs (c) and ^137^Cs (d), in each part of the bamboo shoots collected in Tsukubamirai in Ibaraki Prefecture in April 2012. Error bars indicate measurement deviation.

In April and May 2012, 23 bamboo shoots from *Phyllostachys pubescens* were collected at 10 sites within 41 (Date, Fukushima Prefecture) to 1140 km (Nagasaki, Nagasaki Prefecture) of the Fukushima Daiichi nuclear power plant (**Table 1** **,** **Fig. 1a**). At six sites within 41 (Date, Fukushima Prefecture) to 225 km (Annaka, Gunma Prefecture) of the Fukushima Daiichi nuclear plant, all 14 bamboo shoots contained ^134^Cs and ^137^Cs in the edible parts (**Table 1**). The maximum ^134^Cs and ^137^Cs activities, 15.3 and 21.8 kBq/kg (dry weight), respectively, were measured in the sample from Date, Fukushima Prefecture (41 km from the Fukushima Daiichi nuclear plant) (**Table 1**, Date). The activity concentrations for the bamboo samples correlated with those in the surface soils (upper 2 cm) (**Fig. 2a, b**). In the nine bamboo shoots collected within 440–1140 km of the Fukushima Daiichi nuclear plant, the concentrations were below measurable limits (**Table 1**).

**Table 1:** List of sampling bamboo shoots and their Cs-134 and -137 concentrations in the upper edible part.

Sampling date	Sampling area	Distance to Fukushima Daiichi nuclear power plant (km)	ID	Maximum diameter (cm)	Total length (cm)	Outcrop legth (cm)	Basal part length (cm)	Cs-134 in upper edible part (Bq/kg (DW))	Cs-137 in upper edible part (Bq/kg (DW))
May 9, 2012	Date city	41	A	6	32	10	6	15300±57.4	21800±96.1
May 9, 2012	Date city	41	B	5	30	10	4	1510±21.2	2140±36.4
May 20, 2012	Aizuwakamatsu city	102	A	8	35	10	5	133.5±7.31	201.2±12.6
April 30, 2012	Tsukubamirai city	185	A	12	50	35	5	589.7±11.8	808.2±20.0
April 30, 2012	Tsukubamirai city	185	B	12	35	15	8	668.6±12.1	985.1±20.9
April 30, 2012	Tsukubamirai city	185	C	10	30	10	9	655.2±12.9	927.4±22.2
April 30, 2012	Tsukubamirai city	185	D	10	30	10	3	800.4±16.5	1090±27.6
April 30, 2012	Tsukubamirai city	185	E	8	25	6	3	544.4±15.5	726±25.4
April 30, 2012	Tsukubamirai city	185	F	11	45	20	10	700.1±13.2	1010±22.8
May 12, 2012	Kashiwa city	195	A	4	65	350	-	1140±30.9	1700±52.2
May 12, 2012	Kashiwa city	195	B	8	42	10	12	461±12.5	673.1±21.3
May 12, 2012	Kashiwa city	195	C	5	25	10	4	300±16.9	420±26.1
April 22, 2012	Ichikawai city	215	A	9	28	10	-	694±15.8	997.4±27.3
May 2, 2012	Annaka city	225	A	9	40	20	6	75.4±5.80	119.6±9.27
April 13, 2012	Toyohashi city	440	A	5	16	3	5	<32.0	<33.9
May 4, 2012	Toyohashi city	440	B	6	30	5	5	<33.8	<32.2
May 4, 2012	Toyohashi city	440	C	5	25	5	3	<57.0	<47.8
April 14, 2012	Kizugawa city	555	A	9	30	USS	7	<23.3	<22.6
April 14, 2012	Kizukawa city	555	B	8	24	USS	6	<32.9	<30.3
April 14, 2012	Kizukawa city	555	C	7	20	USS	4	<34.3	<30.8
April 18, 2012	Beppu city	980	A	12	45	35	10	<32.9	<32.8
April 9, 2012	Nagasaki city	1140	A	7	17	2	4	<33.3	<34.0
April 9, 2012	Nagasaki city	1140	B	4	12	2	4	<67.9	<61.7

*USS: under surface soil.

**Figure 3:**
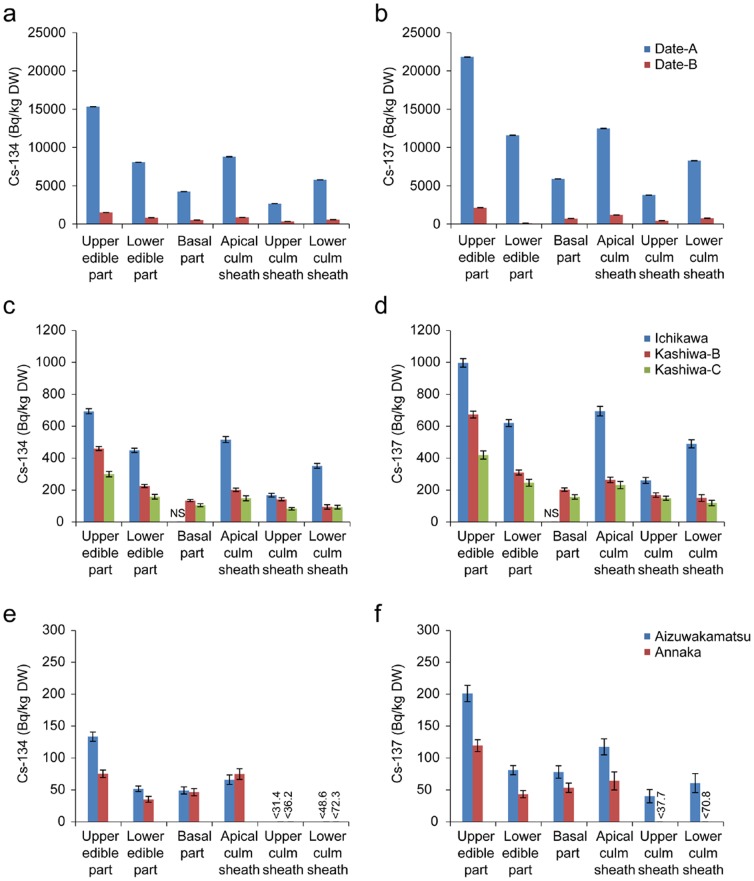
Distribution of radiocesium in bamboo shoots sampled in April and May 2012. (a, b) The radioactive concentrations of radiocesium, ^134^Cs (a) and ^137^Cs (b), in each part of the bamboo shoots collected in Date, Fukushima Prefecture (41 km from Fukushima Daiichi). (c, d) The radioactive concentrations of radiocesium, ^134^Cs (c) and ^137^Cs (d), in each part of the bamboo shoots collected in Ichikawa (215 km from Fukushima Daiichi) and Kashiwa (195 km from Fukushima Daiichi) in Chiba Prefecture. NS indicates not sampled. (e, f) The radioactive concentrations of radiocesium, ^134^Cs (e) and ^137^Cs (f), in each part of the bamboo shoots collected in Aizuwakamatsu in Fukushima Prefecture (102 km from Fukushima Daiichi) and Annaka in Gunma Prefecture (225 km from Fukushima Daiichi). Error bars indicate measurement deviation.

To investigate the radiocesium distribution in the bamboo shoot bodies, the bamboo shoots were separated into six parts (**Fig. 1b**). The moisture contents of each part were quite similar, being around 85% (**Fig. S1**). The upper edible part and the apical culm sheath, which are both inner tip parts, tended to have higher radiocesium concentrations in every contaminated sample (**Fig. 2c, d** **,** **Fig. 3**).

**Figure 4:**
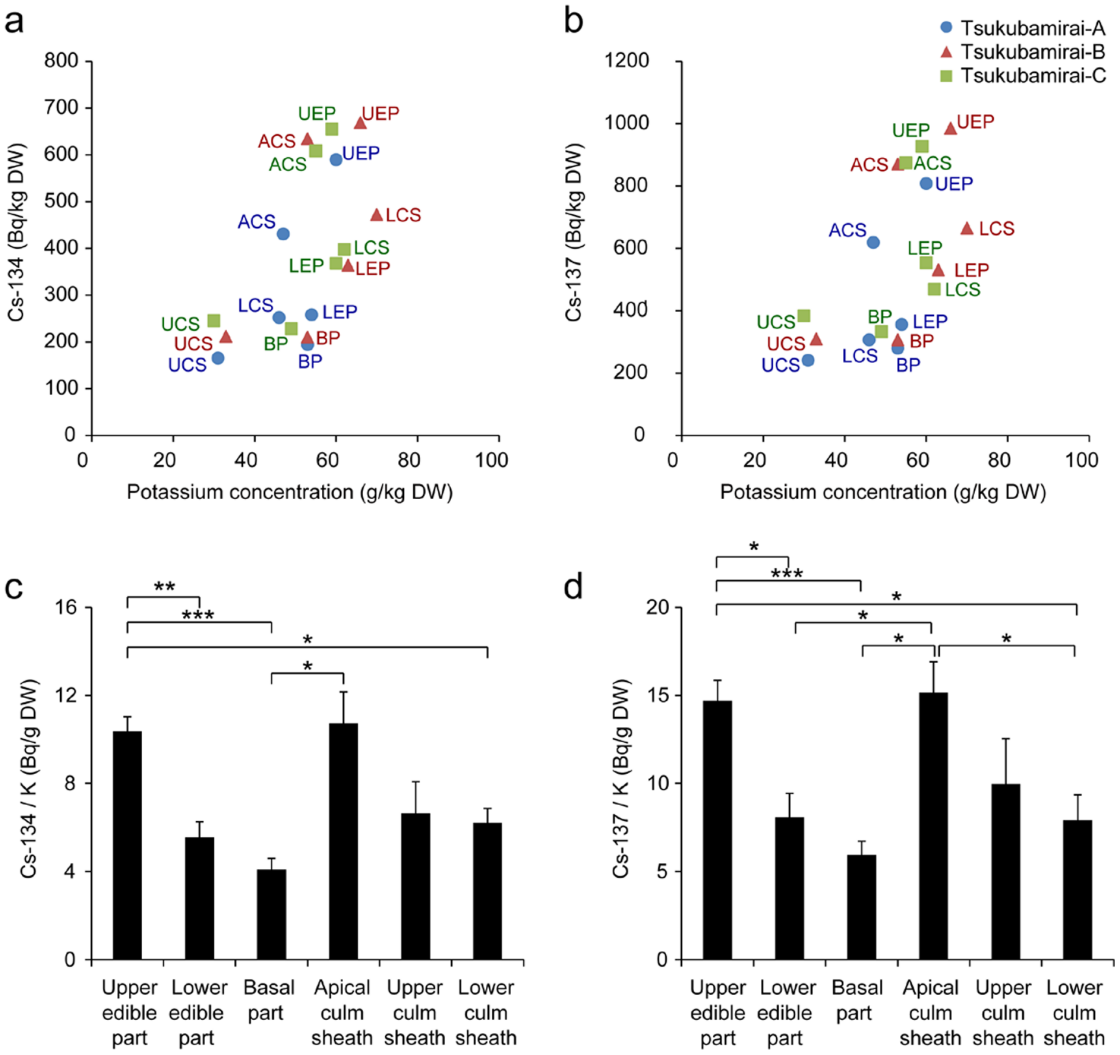
Comparison of radiocesium and potassium in each part of the bamboo shoots of *Phyllostachys pubescens*. (a, b) Scatter plots of the radioactive concentrations of radiocesium, ^134^Cs (a) and ^137^Cs (b), and the potassium concentrations. UEP, LEP, BP, ACS, UCS, LCS indicate the upper edible part, lower edible part, basal part, apical culm sheath, upper culm sheath and lower culm sheath, respectively. (c, d) The mean ratio of the radioactive concentrations of radiocesium, ^134^Cs (c) and ^137^Cs (d), to the potassium concentrations. Error bars show the standard deviation for three separate bamboo shoots in Tsukubamirai as shown in (a, b). Significance was determined using Welch's two-sample test. *p*-value *<0.01, **<0.003, ***<0.001.

Next, the relationship between concentrations of potassium and radiocesium was examined because of their chemical similarities as alkali elements; three samples from Tsukubamirai, Ibaraki Prefecture, were investigated. The ratio of radiocesium to potassium concentrations (**Fig. 4**) as well as the absolute radiocesium concentrations (**Fig. 2c, d**) tended to be higher in the inner tip parts of the bamboo plants. Cesium is an alkali element, and the nuclear accident-derived radiocesium has been assumed to be taken up by plants in the same manner as potassium [16]. However, our results suggest that the correlation between radiocesium and potassium concentrations was somewhat unique to each specific plant tissue, at least in the bamboo shoots examined in this work.

**Figure 5:**
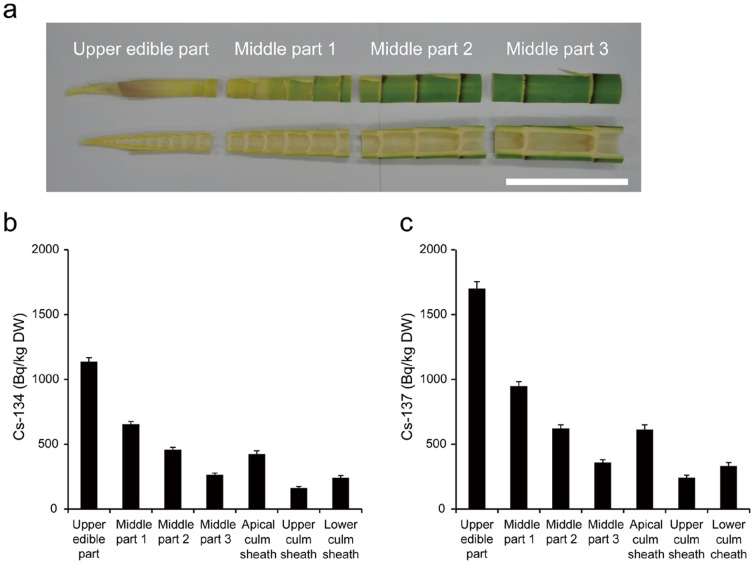
Radiocesium contamination in bamboo shoots of *Phyllostachys pubescens* with an outcrop length of 3.5 m. (a) The separated tall bamboo shoot collected in Kashiwa in Chiba Prefecture (195 km from Fukushima Daiichi). The stripped bamboo shoots were cut at 30 cm intervals from the tip, separating the upper edible part, and the middle parts 1, 2 and 3. Scale bar indicates 30 cm. (b, c). The radioactive concentrations of radiocesium, ^134^Cs (b) and ^137^Cs (c), in each part of the bamboo shoots shown in (a). Error bars show one standard deviation.

**Figure 6:**
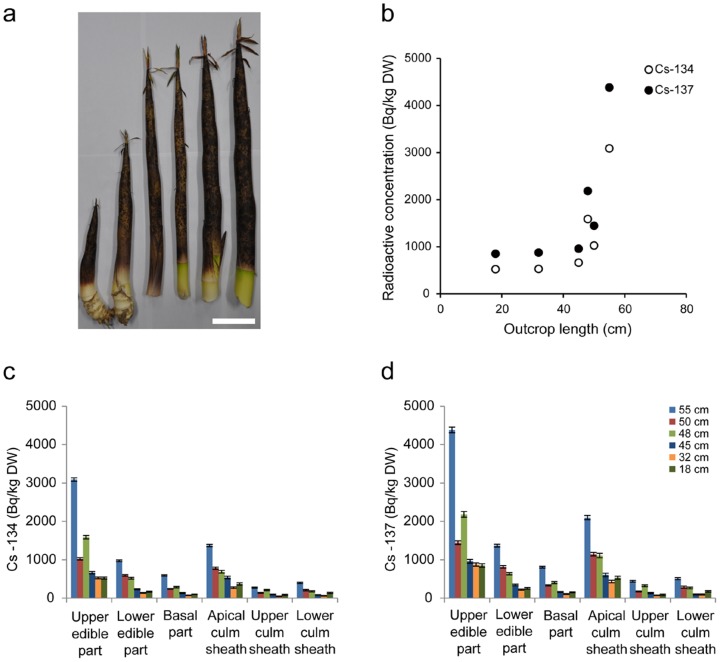
Relationship between outcrop lengths and the radiocesium concentrations in bamboo shoots of *Phyllostachys bambusoides* Sieb. Et Zucc. (a) The collected *Phyllostachys bambusoides* bamboo shoots. Scale bar indicates 10 cm. (b) The scatter plot of the radioactive concentrations of radiocesium in tip parts and the outcrop length. (c, d) The radioactive concentrations of radiocesium, ^134^Cs (c) and ^137^Cs (d), in each part of the bamboo shoots shown in (a). Error bars show measurement deviation.

In the tall bamboo shoots of *P. pubescens* (3.5 m in height), the upper edible part showed the highest radiocesium concentrations (**Fig. 5**). Interestingly, the absolute radiocesium concentrations were higher than those of short bamboo shoots (10 cm) at the same sampling site (**Table 1**, Kashiwa, Chiba Prefecture). Unfortunately, because of its large size, *P. pubescens* was unsuitable for sampling and handling to investigate the relationship between radiocesium concentrations and bamboo shoot growth. However, a similar tendency was observed in another bamboo species, *Phyllostachys bambusoides* Sieb. Et Zucc., that produces small edible shoots. At the same sampling site, radiocesium activities tended to increase with the outcrop length (**Fig. 6a, b**) with higher concentrations in the upper edible parts (**Fig. 6c, d**). This finding suggested that the bamboo shoots continued to take up radiocesium isotopes and accumulate the element in the inner tip parts during growth, at least in *P. bambusoides*.

## Discussion

In this study, we report the Fukushima nuclear accident-derived radiocesium concentrations in bamboo shoots for the period in 2012. The findings can be summarized by two main points: 1) inner tip parts, such as the upper edible part, had higher radiocesium activity; and 2) tall bamboo shoots showed higher radiocesium concentrations than short bamboo shoots.

In our bamboo shoot samples, inner tip parts showed higher potassium concentrations than hardened parts (**Fig. 4a, b**). This finding is consistent with previous reports that show a decrease in potassium concentrations with bamboo age [17]. Because potassium and cesium are both alkali elements, it is reasonable to expect that radiocesium concentrations will also be higher in the inner tip parts (**Fig. 2c–f** **,** **3** **,** **5b, c** **,** **6c, d**). However, the concentration ratios of radiocesium to potassium found possibly suggest radiocesium accumulation in the inner tip parts (**Fig. 4c, d**). At this stage, it is therefore sensible to avoid eating the edible tips of contaminated bamboo shoot to lessen internal exposure to radiocesium.

Tall bamboo shoots showed higher radiocesium concentrations, at least in *P. bambusoides* (**Fig. 6**), suggesting that absorbed and entrapped radiocesium in above- and below-ground biomass did migrate to rapid growing bamboo shoots. Similar translocations of the Fukushima accident-derived radiocesium to new plant growth including, shoots, leaves, and fruits, were previously reported for several herbaceous and woody plants [6]. To reduce the radiocesium contamination in bamboo shoots, the radiocesium absorption pathway in bamboo plants must be identified. There are two possible routes for accumulation of radiocesium in bamboo; one is root absorption from contaminated soils, and the other is above-ground surface absorption from attached microgranules [15]. Previous measurements on the depth distributions of radiocesium showed that radiocesium largely existed within the top 5 cm of soil but the element was also detected in deeper horizons (below 20 cm), suggesting a deeper penetration into the soil profile via a preferential penetration path (e.g., worm holes) [18]. Because the underground stem-root system of bamboo mainly exists in the soil layers, we cannot eliminate the possibility of root absorption. However, we previously reported higher radiocesium activities in mature leaves than in young ones in bamboo plants 25–195 km from the Fukushima Daiichi nuclear plant 4 months after the nuclear accident [7], suggesting direct deposition of radiocesium fallout onto mature leaves. In addition, after the Chernobyl nuclear accident, studies of thyme plants (*Origanum vulgare* L.) in Turkey suggested the importance of foliar absorption of radiocesium rather than root uptake [19]. Recently, following the Fukushima nuclear accident, granular radioactive spots have been observed on bamboo shoot skin as well as in the leaf litter [15]. At present, therefore, we cannot rule out the contribution of either pathway to radiocesium contamination in bamboo plants.

In the future, an investigation of annual changes in radiocesium concentrations in bamboo shoots will be important. In May 2013, we sampled six bamboo shoots from the same bamboo bush in Tsukubamirai (185 km from the Fukushima Daiichi nuclear plant) (**Table S1**). ^134^Cs and ^137^Cs were detected in all samples, and higher radiocesium concentrations were detected in the inner tip parts of all samples, the absolute ^137^Cs concentrations in 2013 being roughly half of those in 2012 (**Fig. S2**). Because of its long half-life (30 years), natural nuclear decay does not explain this result. Actually, radiocesium concentrations of the surface soil were comparable to those in 2012 (**Table S2**). Unfortunately, at present, we cannot explain why the radiocesium concentrations in shoots in 2013 were lower than expected. In 2013, the sampled bamboo bush yielded a relatively poor harvest with fewer emerging shoots and with a slower growth rate than in 2012. Although a causal relationship between the lower radiocesium concentrations and the poor harvest is unproven, the reduced shoot growth may have been owed to the decreased radiocesium concentrations in 2013. To assess the annual changes in radiocesium contamination in bamboo shoots, further long-term investigations are needed. Additional works are planned for interannual monitoring for radiocesium distribution with in-plant replication.

## Supporting Information

Figure S1 **Moisture contents in each part of sampled bamboo shoots of Phyllostachys pubescens sampled in 2012.** NS indicates not sampled.
(TIF)

Figure S2 **Radiocesium contamination in bamboo shoots of** ***Phyllostachys pubescens*** **.** The radioactive concentrations of radiocesium, ^134^Cs (a) and ^137^Cs (b), in each part of the bamboo shoots collected in Tsukubamirai city in Ibaraki Prefecture in May 2013. Error bars show measurement deviation.
(TIF)

Table S1 List of bamboo shoots sampled in 2013 and their radiocesium concentrations in the upper edible part.
(DOCX)

Table S2 Radiocesium concentrations in surface soil samples (above 2 cm) in Tsukubamirai city (185 km from Fukushima Daiichi).
(DOCX)
