# Supplementary figures and images for: Radiocesium Distribution in Bamboo Shoots after the Fukushima Nuclear Accident
Source: PLoS One. 2014 May 15;9(5):e97659. doi: 10.1371/journal.pone.0097659 (PMC4022665; doi:10.1371/journal.pone.0097659)

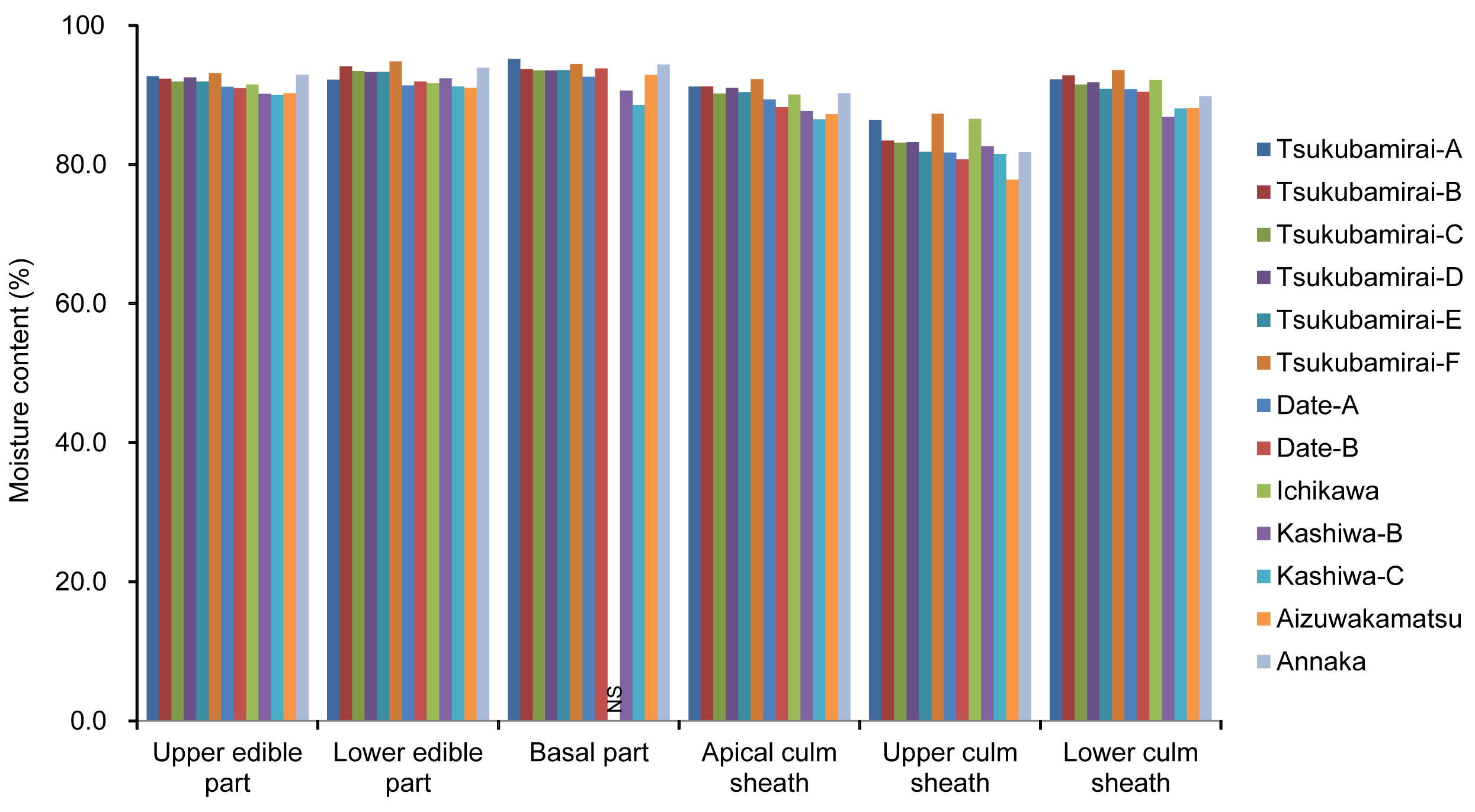

Supplement: Figure S1 — Moisture contents in each part of sampled bamboo shoots of Phyllostachys pubescens sampled in 2012. NS indicates not sampled. (TIF) [file pone.0097659.s001.tif]

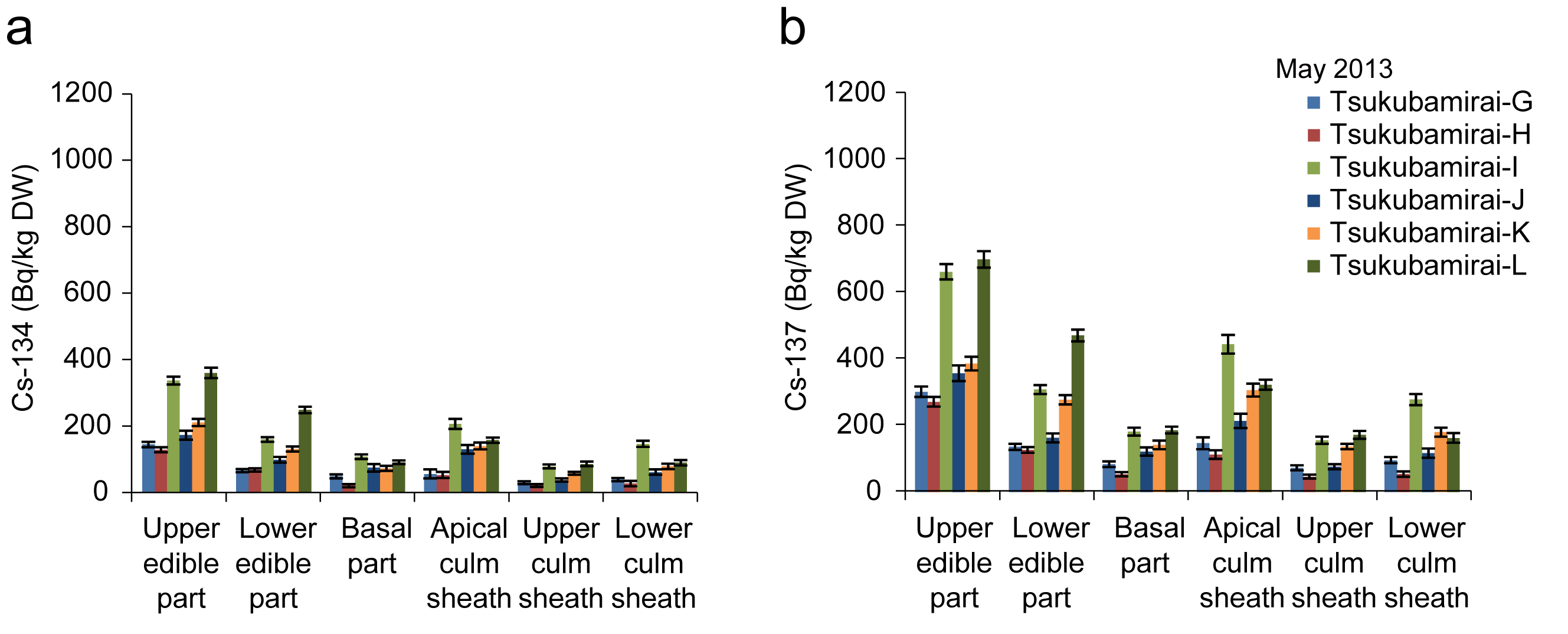

Supplement: Figure S2 — Radiocesium contamination in bamboo shoots of Phyllostachys pubescens . The radioactive concentrations of radiocesium, 134Cs (a) and 137Cs (b), in each part of the bamboo shoots collected in Tsukubamirai city in Ibaraki Prefecture in May 2013. Error bars show measurement deviation. (TIF) [file pone.0097659.s002.tif]
