# Supplementary material for: Radiocesium Distribution in Bamboo Shoots after the Fukushima Nuclear Accident
Source: PLoS One. 2014 May 15;9(5):e97659. doi: 10.1371/journal.pone.0097659 (PMC4022665; doi:10.1371/journal.pone.0097659)
Supplement: Table S1 — List of bamboo shoots sampled in 2013 and their radiocesium concentrations in the upper edible part. (DOCX) [file pone.0097659.s003.docx]

**Table S1.** List of bamboo shoots sampled in 2013 and their radiocesium concentrations in the upper edible part.
