# Supplementary material for: Radiocesium Distribution in Bamboo Shoots after the Fukushima Nuclear Accident
Source: PLoS One. 2014 May 15;9(5):e97659. doi: 10.1371/journal.pone.0097659 (PMC4022665; doi:10.1371/journal.pone.0097659)
Supplement: Table S2 — Radiocesium concentrations in surface soil samples (above 2 cm) in Tsukubamirai city (185 km from Fukushima Daiichi). (DOCX) [file pone.0097659.s004.docx]

**Table S2.** Radiocesium concentrations in surface soil samples (above 2 cm) in Tsukubamirai city (185 km from Fukushima Daiichi).
